# Cardiac Optical Mapping in Situ in Swine Models: A View of the Current Situation

**DOI:** 10.3390/medicina56110620

**Published:** 2020-11-18

**Authors:** Irma Martišienė, Regina Mačianskienė, Rimantas Benetis, Jonas Jurevičius

**Affiliations:** 1 Institute of Cardiology, Lithuanian University of Health Sciences, LT-50161 Kaunas, Lithuania; irma.martisiene@lsmuni.lt (I.M.); regina.macianskiene@lsmuni.lt (R.M.); rimantas.benetis@lsmuni.lt (R.B.); 2 Department of Cardiac, Thoracic and Vascular Surgery, Hospital of Lithuanian University of Health Sciences Kauno Klinikos, Lithuanian University of Health Sciences, LT-50161 Kaunas, Lithuania

**Keywords:** optical mapping, pig heart, swine model in situ

## Abstract

Optical mapping is recognized as a promising tool for the registration of electrical activity in the heart. Most cardiac optical mapping experiments are performed in ex vivo isolated heart models. However, the electrophysiological properties of the heart are highly influenced by the autonomic nervous system as well as humoral regulation; therefore, in vivo investigations of heart activity in large animals are definitely preferred. Furthermore, such investigations can be considered the last step before clinical application. Recently, two comprehensive studies have examined optical mapping approaches for pig hearts in situ (in vivo), likely advancing the methodological capacity to perform complex electrophysiological investigations of the heart. Both studies had the same aim, i.e., to develop high-spatiotemporal-resolution optical mapping suitable for registration of electrical activity of pig heart in situ, but the methods chosen were different. In this brief review, we analyse and compare the results of recent studies and discuss their translational potential for in situ cardiac optical mapping applications in large animals. We focus on the modes of blood circulation that are employed, the use of different voltage-sensitive dyes and their loading procedures, and ways of eliminating contraction artefacts. Finally, we evaluate the possible scenarios for optical mapping (OM) application in large animals in situ and infer which scenario is optimal.

## 1. Introduction

The heart is one of the organs of which its electrophysiological properties are closely related to the autonomic nervous system as well as humoral regulation. In the last few decades, there has been constant interest in optical mapping (OM) as a prospective tool for the registration of electrical activity of the heart. Most cardiac OM experiments are performed ex vivo in isolated heart models. However, cardiac experimentation in live organisms creates conditions that are close to native ones and allows one to perform complex electrophysiological investigations of the heart. It should be noted that different authors use different terms, in situ or in vivo, to define experimentation conditions in live organisms. The first work on registration of cardiac electrical signals by optical fibres in vivo was performed in a canine model [1]. A much later study reported multiparametric imaging of the rat heart in situ under cardiopulmonary bypass [2]. However, due to possible technical challenges, the development of OM in large animal models in situ has long remained uninvestigated. Very recently, Lee et al. [3] and our group [4] published studies of OM approaches for the pig heart in situ (in vivo), likely advancing the methodological capacity to perform complex electrophysiological investigations of the heart. Both studies had the same aim, i.e., to develop high-spatiotemporal-resolution OM suitable for registering the electrical activity of the pig heart in situ, but different methods were chosen. To date, there have been no other studies on the application of high-spatiotemporal-resolution OM in swine models in situ. This encouraged us to analyse and compare the recent results and to discuss their translational potential for in situ cardiac OM applications in large animals.

In general, it is accepted that the swine model is valuable in pre-clinical research because of its similarities to humans, for example, in terms of its cardiovascular system and body weight [5,6]. In situ cardiac OM in swine models have two major aspects that should be assessed: (1) studies in situ on large animal models allow conditions to be created that are close to the usual conditions for humans; (2) these animal studies can be considered the last step before the clinical application of OM. However, large animal models are challenging and require various factors and conditions to be considered. Working with large animals requires specific equipment and large amounts of chemicals. Moreover, longer gestation time and lifespan, higher maintenance costs, harder handling, etc. contribute to the limitations of large animal models [6].

## 2. OM in Situ under Physiological and Artificial Blood Circulation

Electrophysiological studies in an open-chest heart model can be performed in two ways, i.e., under physiological or artificial blood circulation. The main limiting factor of high-spatiotemporal-resolution OM is the distortion of optically recorded electrical signals by motion artefacts. The choice of blood circulation type determines which options are available for contraction artefact elimination, which is crucial for OM quality. Lee and co-authors present two ways of overcoming the impact of cardiac contraction on optically registered electrical activity in a freely contracting pig heart in vivo under physiological blood circulation: excitation ratiometry and optical-fibre recordings [3]. In their study, excitation ratiometry with a single-camera system was applied under conditions that are associated with reduction of heart contraction, i.e., high stimulation frequency (300 ms cycle length) or ventricular fibrillation. However, a previous investigation of the force-frequency relationship in pig ventricular myocardium in vivo showed that contraction force reaches its maximum at a stimulation frequency of approximately 300 ms [7]. This suggests that pacing with a 300 ms cycle length cannot be considered high stimulation frequency to reduce contraction force of a pig heart. Nevertheless, independent of heart contraction force, ratiometric mapping under such conditions could be suitable for producing activation maps, but not sufficient for constructing repolarization or action potential duration (APD) maps [8]. In addition, in the study by Lee et al. [3], APD maps were constructed using optical-fibre fluorescence recordings. Moreover, recordings from only 16 points in a 12 × 12 mm area of the heart cannot be considered high spatial resolution.

The study by our group presents data obtained by using a custom-made frame (glued to the epicardial surface in a special procedure) that enabled us to record optical signals from a 40 × 40 mm area of the pig heart in situ under physiological blood circulation with applied stimulation (500 ms cycle length) or ventricular fibrillation. However, our data revealed that mechanical motion arrest using a frame partially restricts the field of view and does not fully eliminate motion artefacts [4]. Thus, OM under physiological blood circulation, for which the heart must function as a pump, is easy to create in principle, but complicated to apply for qualitative registration at high spatiotemporal resolution.

Artificial blood perfusion, a routine procedure applied in clinical open-heart surgery, ensures controlled conditions and allows an electromechanical uncoupler to be applied. As our study suggests, it is a promising mode of circulation for the registration of cardiac electrical activity using OM [4]. However, artificial blood circulation requires additional equipment and advanced specialists. Moreover, the use of uncouplers, such as blebbistatin, may induce side effects on electrophysiological parameters of the heart [3,9]. Our study [4], in agreement with others [10,11,12], revealed no significant effect of blebbistatin on the electrophysiological parameters of pig hearts in situ. However, in terms of clinical application, the lack of detailed information on the toxicity of this uncoupler is still a limiting factor in its usage for eliminating contraction artefacts during OM under artificial blood circulation. It would be worthwhile to search for other alternatives. Depending on the purpose of the study, various drugs can be used. For instance, in order to investigate the pattern of electrical impulse propagation in the heart by OM under artificial blood circulation, L-type Ca^2+^ channel antagonists, such as diltiazem or verapamil, could be exploited. These agents are commonly used in clinical practice as class IV antiarrhythmic drugs; they are rapidly metabolized and therefore could serve as safe temporary contraction suppressors.

## 3. Voltage-Sensitive Dyes in OM in Situ

Different voltage-sensitive dyes were used in our study and that of Lee et al.: di-4-ANBDQBS [3,4], di-4-ANEQ(F)PTEA [3], and Cardiogreen [4]. Di-4-ANBDQBS is a near-infrared fluorescent dye that has been commonly used in OM studies since the demonstration of its high voltage sensitivity and suitability for use in the blood-perfused myocardium [13]. Di-4-ANEQ(F)PTEA is a fluorinated near-infrared voltage-sensitive dye with improved characteristics such as solubility and photostability [14]. In the study by Lee et al. [3], a great deal of attention was devoted to comparing the dyes di-4-ANBDQBS and di-4-ANEQ(F)PTEA. The authors did not report any significant differences in the quality of optical signals obtained from different dyes; however, they found that heart staining (loading) in vivo with di-4-ANEQ(F)PTEA was easier than staining with di-4-ANBDQBS. A study of the toxicity of both dyes, di-4-ANBDQBS and di-4-ANEQ(F)PTEA, showed that neither had a significant effect on pig vital organs, which is a promising sign for their clinical application prospects [3]. However, these dyes are not approved by the FDA as safe for clinical use. Recently, it has been shown that Cardiogreen, which is currently the only FDA-approved fluorescent dye and has long been widely used to visualize vessels in clinical settings, has voltage sensitivity [15] and can be used in OM to record the electrical activity of the heart [16,17]. However, Cardiogreen has low fractional fluorescence, which is its main disadvantage as a fluorescent voltage-sensitive dye in OM of large animal hearts in situ.

In addition, the two studies reported different strategies for fluorescent dye loading of pig hearts in situ. Lee et al. [3] performed staining of the heart via a catheter that was introduced into the proximal left anterior descending artery in the presence and absence of coronary blood flow under physiological blood circulation. The authors found that dye loading in the presence of coronary blood perfusion in vivo was a less dangerous intervention for the animal than loading under conditions of temporal stopped coronary blood flow [3]. In our study, staining of the heart was carried out by introducing the dye through an aortic root cannula during a temporary blockade of blood perfusion to the body, which was created by clamping the aorta below the arterial return cannula. Under physiological blood circulation, the dye was introduced under applied pressure, while under artificial blood circulation, the dye was directly injected through the infusion system [4]. The injection of the dye straight into the coronary blood flow through the catheter requires additional equipment (such as an X-ray machine) and special skills, whereas artificial blood circulation provides convenient conditions for staining the heart with fluorescent dye, which can be introduced simultaneously with other agents.

## 4. Motion Artefact Elimination in OM in Situ

In principle, the two studies demonstrate OM of the pig heart in situ using different methodologies: one used excitation ratiometry in the freely contracting heart and optical-fibre recordings from 16 points [3], whereas the other applied mechanical immobilization and chemical arrest of heart contraction [4]. These distinct methods have their own advantages and disadvantages that must be considered (Table 1).

Application of ratiometric analysis in freely contracting hearts has limited potential. Excitation ratiometry can reduce the influence of heart contraction movements, but it cannot eliminate the effects of the twisting movement that a freely contracting heart makes when not immobilized. It has been demonstrated that heart immobilization or mathematical data processing should be used to eliminate the effects of twisting [11,18]. Recordings obtained using excitation ratiometry in freely contracting hearts can help track the propagation of excitation in the heart; however, they are not sufficient to construct maps of APD [8]. Fluorescence ratiometry is commonly applied to eliminate motion artefacts during OM ex vivo. In our study, fluorescence ratiometry was used for processing optical signals obtained under artificial blood circulation in the presence of blebbistatin. Data have shown that the ratio of two signals obtained at different excitation wavelengths may hide interlayer differences in the myocardium, such as different durations of action potential upstroke or alternans formation at certain layers [4]. Obviously, excitation ratiometry, where two light-emitting diodes (LEDs) of different excitation wavelengths are used in alternation during one action potential, is easy to apply. However, different depths of penetration into the myocardium may be a limiting factor in certain investigations, such as studies of arrhythmia formation. To overcome this, one can use emission ratiometry or combined excitation/emission ratiometry. However, such techniques are more technically complex and require additional equipment such as two cameras and a splitter. Moreover, ratiometry can be applied only with standard voltage-sensitive dyes. Ratiometry of Cardiogreen optical signals is difficult to achieve as they contain fast and slow components and their characteristics depend on the excitation/emission wavelengths [16,17].

The use of an optical-fibre array (4 × 4) allows the electrical activity of the heart to be recorded in vivo under physiological blood circulation [3]. However, in large animals or humans, the main disadvantage of this method is the small mapping area, which does not permit high-spatial-resolution mapping. Obviously, the mapping area can be increased by using an array with a larger number of optical fibres. On the other hand, in order to use optrodes in vivo under physiological blood circulation, pressure must be applied to the heart to minimize contraction and twisting. Excessive pressure may be harmful to the heart tissue and this fact is one of the disadvantages of using optrodes under physiological blood circulation.

Mechanical immobilization of the heart using a custom-made frame or Octopus tissue stabilizer is another option for OM of the freely contracting heart [4]. The use of such mechanical immobilization significantly reduces contraction artefacts in optical signals. Additionally, specific fixation of the frame and stabilizer, i.e., gentle lifting of the heart by an additionally fixed bar connected to a manipulator, minimizes motion caused by lung ventilation. Overall, both types of mechanical immobilization restrict the mapping area. Furthermore, such immobilization of the heart could be conducive to use of ratiometry.

In our study, chemical arrest of heart contractions under artificial blood circulation was induced by injecting blebbistatin together with fluorescent dye directly into the aorta through an inserted cannula [4]. The effect of this uncoupler was tested in both studies, but the methods and results were different. The effect of blebbistatin on the conduction velocity and APD of pig hearts was tested using an optical-fibre array ex vivo [3] and on the electrographic parameters of pig hearts, which reflect APD, using electrogram electrodes in situ [4]. Lee et al. [3] found that blebbistatin slightly increased the APD and decreased the conduction velocity of isolated Langendorff-perfused pig hearts, while our study showed no significant effect on the electrographic parameters of pig hearts in situ. The increased APD reported by Lee et al. could be related to heart relaxation and to the reduction of possible ischaemic processes due to Langendorff perfusion [19]. Additionally, ischaemia can be evoked by applying pressure to the heart, which is necessary when using an optrode. Obviously, to investigate the effect of the electromechanical uncoupler on the pig heart in situ, it is important to choose the correct registration pattern, one that is suitable for recording the electrical activity of the myocardium in both freely contracting and stopped conditions. Therefore, the effect of blebbistatin in our study was determined in situ using electrograms; the tested parameters reflecting APD showed no change or even tended to decrease [4]. Principally, our data are the first to demonstrate the effect of blebbistatin on pig hearts in situ and can hardly be compared with the many datasets that have been collected in ex vivo conditions.

## 5. Conclusions

The development of in situ high-spatiotemporal-resolution OM in swine models is a significant step towards comprehensive cardiac electrophysiological investigations in the whole organism and, consequently, towards its application in clinics. In general, OM in humans could be applied in the detailed characterization of propagation of cardiac electrical impulses along with detection of stable ectopic foci or zones with altered excitability, which is crucial for the determination of arrhythmogenesis. As it was demonstrated in the studies, OM enables registration of the pathways of electrical impulse propagation during ventricular fibrillation [3,4] or ischemia [4]. Moreover, the high-spatiotemporal-resolution registration of ventricular reentry pathways may help to reveal the origin of obstacles, i.e., functional or morphological [4]. Also, registration of changes of activation time and APD may help to determine the ischemic zone during ischemia [4].

Current research data on OM of the pig heart in situ present different methods of contraction artefact elimination that depend on the mode of blood circulation. In addition, the use of different voltage-sensitive dyes and loading methods is examined. From our point of view, based on the results of the two reviewed studies, it can be assumed that the best (optimal) scenario in which to apply OM in large animals in situ could be chemical arrest of heart contraction under artificial blood circulation. However, it should be considered that the use of chemical uncouplers to eliminate heart motion artefacts places the heart under non-physiological conditions where cardiac metabolism is reduced due to the absence of contraction. The other option is OM in situ using mechanical heart immobilization, such as frame, which likely allows ratiometry to be applied more effectively than in chemical arrest. This option for in situ OM maintains conditions closer to a physiological state but significantly limits the spatial mapping of the heart.

## Figures and Tables

**Table 1:** Advantages and disadvantages of methods to overcome motion artefact used in cardiac optical mapping in situ in swine models.

Cardiac Optical Mapping in situ in Swine Models				
Blood Circulation Type	Physiological			Artificial
Method to Overcome Motion Artefact	Array of Optical Fibre [3]	Mechanical Immobilization [4]	Excitation Ratiometry [3,4]	Chemical Arrest [4]
Advantages	Registration without excitation-contraction uncouplers			High spatiotemporal resolution
	Construction of activation time and action potential duration (APD) maps		Construction of activation time maps	Construction of activation time, repolarization, conduction velocity and APD maps
Disadvantages	Low spatial resolution	Restricted mapping area	Insufficient signal to construct APD and repolarization maps	Need to use excitation-contraction uncouplers
	Possible ischemic damage because of necessary pressing of the heart to minimize contraction and twisting	Need to use ratiometry for residual motion artifact elimination	High motion artifact without additional heart immobilization	No possibility to record excitation-contraction function
